# Delay in Attention to Casualties

**Published:** 1907-06-01

**Authors:** 


### RESIDENT MEDICAL OFFICERS' DEPARTMENT.

[Contributions to this Column are invited, and, if accepted, will be paid for.]

### DELAY IN ATTENTION TO CASUALTIES.

Hospital patients now and then complain that they are kept waiting for long periods of time before they receive attention from the medical officer on duty. Sometimes these complaints reach the ears of the hospital authorities, and the house physician or house surgeon is in consequence haled before the Board. Being unable, perhaps, to recall the exact details of circumstances of a week ago, he has no definite excuse to offer, and is accordingly censured—more often than not quite unjustly.

The medical officer on duty in most hospitals is expressly ordered to attend to all casualty cases without delay, and to consider this paramount to any other work upon which he may be engaged at the time. In theory this is only right; but in practice it is not always feasible, as anyone who has worked in hospital knows perfectly well. There are many duties which cannot justifiably be left half-finished, except to attend to cases of extreme urgency. The house surgeon on duty has all his ordinary work to do, and, were he to obey every summons from the casualty department the instant he received it, and without regard to whatever else he might be occupied with at the moment, the condition of his wards and his patients and of the nursing staff would become intolerable—a chaos of worry and flurry, of half-finished dressings, of half-given orders, and of examinations and minor operations interrupted at critical moments.

If it is really essential that all casualty patients should be seen immediately on arrival, then the resident staff of every hospital must be so increased in number that one medical officer may always be on duty, with nothing else to do, and with proper accommodation for kicking his heels when business is slack. There is no other way of making absolutely certain that each patient is seen without delay, irrespective of whether his complaint is trivial or urgent.

As a matter of fact the present system on the whole works admirably, and it is the rarest thing for any case of the least urgency to be kept waiting long enough to cause harm. Hospital porters are generally very shrewd fellows, and they seldom fail to secure the prompt attention of the house surgeon to an urgent case; and when they are in doubt they are far more likely to over-colour the gravity of the patient's appearance than to make too light of it.

The patients who pester kind-hearted clergymen and hospital subscribers until the latter lodge complaints of delay with the governors are very often not the necessitous poor, but members of that class of parasites who can afford to pay for medical advice, to whom we devoted an article in a previous issue. Those who abuse hospitals in one sense are nearly always the first to abuse them in another. The genuinely poor are, in the writer's experience, much more patient, considerate and grateful than are those who seek free treatment to which they are not entitled.

